# An Investigation Into the Relationship Between Onset Age of Musical Lessons and Levels of Sociability in Childhood

**DOI:** 10.3389/fpsyg.2018.02244

**Published:** 2018-11-26

**Authors:** Satoshi Kawase, Jun’ichi Ogawa, Satoshi Obata, Takeshi Hirano

**Affiliations:** ^1^Yamaha Music Foundation, Tokyo, Japan; ^2^Graduate School of Human Sciences, Osaka University, Osaka, Japan; ^3^Graduate School of Information Systems, The University of Electro-Communications, Tokyo, Japan; ^4^College of Performing and Visual Arts, J. F. Oberlin University, Tokyo, Japan

**Keywords:** group music lesson, sociability, empathy, critical period, infant, joint music

## Abstract

Previous studies have suggested that musical training in childhood is beneficial for sociability. However, it remains unclear how age of onset of group music lessons is associated with the late sociability of children from a long-term perspective. This study investigated associations between group music lessons conducted at a music school and children’s levels of sociability by focusing on the age of onset of the lessons. We conducted a survey of 276 children aged 4–5 years (*M* = 58.5 months) and 6–7 years (*M* = 82.7 months) who commenced music lessons at ages 1, 2, 4, and 6 years. We found that (1) the empathy scores of children aged 6–7 years who began lessons when 1-year-old were greater than those who began lessons when 4-years-old, (2) the communication scores of children aged 4–5 years who began lessons when 1-year-old were greater than those who began lessons when older than 1 year, and (3) the empathy and extraversion scores were high in those aged 6–7 years who began lessons in that age range. The results suggest that early onset of music lessons could positively influence children’s sociability; in contrast, after about age 7 years, children who already had high sociability may be more inclined to select group music lessons. By focusing on the impact of regular group music lessons from a very young age on later levels of sociability, these results further elucidate the effects of musical lessons. In sum, participation in group music lessons 2–4 times per month can be effective social training for very young children and foster their later sociability.

## Introduction

Previous studies have suggested that music activities enhance children’s sociability (as reviewed in Ilari, 2016). Even for infants, musical activities can enhance social skills. Compared with passive music activity (listening to music only), active music lessons for 6-month-old infants facilitated communication and social interactions with parents (Gerry et al., 2012). In those active lessons, infants received Suzuki Early Childhood Education once per week and were encouraged to repeat the songs and rhymes every day at home. Regarding older children, 10 months of music lessons for children with a mean age of 9 years reinforced their sympathy and prosocial behavior (Schellenberg et al., 2015). These findings suggest that early participation in active music lesson can enhance sociability.

As a review of the influence of music lessons (e.g., self-esteem; Hallam, 2010) has suggested, music participation in school also enhances sociability. Musical instrument teachers reported notable improvements in social skills in their students (Hallam and Prince, 2000). An intervention for 12-year-olds that included group drumming (DRUMBEAT) enhanced self-esteem and evaluations by teachers of items such as children’s relationships with peers (Wood et al., 2013). Nevertheless, the findings focused only on the effects of musical activities during a relatively short period.

Group music activity has also been found to be related to sociability (as reviewed in Hallam, 2010). Synchronization among group members is suggested to be the key to promoting sociability by group music activity. Kirschner and Tomasello (2010) examined the relationship between collaborative music activity and helping behavior in 4-year-old children who engaged in 3 min of musical collaboration and suggested that the children who synchronized with peers showed more helping behavior than did the children who only talked with peers. Similarly, empathy levels of students aged 10 years increased after 1 year of music-group interactions, which included providing entrainment or communicating with others, compared with those who did not receive such music-group interactions (Rabinowitch et al., 2012). Furthermore, 14-month-old children’s spontaneous helping behavior was promoted when the experimenters synchronously moved to music as the children listened (Cirelli et al., 2014). Considering these findings, synchronization with other members during group music lessons can be associated with children’s sociability. Specifically, given that group music lessons including multiple interactions among a teacher, peers, and parents requires more varying types of social interactions than does one-to-one lessons, such social interactions in group music lessons may influence children’s sociability.

In non-musical contexts, synchronization has been found to facilitate prosociality (Carpenter et al., 2013; Cirelli et al., 2014; Keller et al., 2014), such as cooperative skills (Valdesolo et al., 2010), compassion and altruistic behavior (Valdesolo and DeSteno, 2011), and empathy (Behrends et al., 2012). Considering these findings, synchronization in group music lessons is likely to be related to sociability. This assumption is also grounded in the finding that joint music performance requires social skills (Keller, 2014; Kawase, 2015; Volpe et al., 2016).

Benefits of parent–child musical activity have also been reported (reviewed in Edwards and Abad, 2016), including attachment (Creighton, 2011; Edwards, 2011). Promotion of attachment by musical co-activity can be explained by the finding that mother–infant synchronization facilitates attachment (e.g., Isabella and Belsky, 1991). Since attachment is associated with children’s social-cognitive skills (reviewed in Thompson, 2008), we hypothesized associations between levels of sociability and group music lessons in which parents help their children and peripherally engage in the same activities as their children. Indeed, a study showed that group music lessons for children, which were similar to the lessons in the present study, improved accompanying parents’ mood (Kawase and Ogawa, 2018). In that study, parent–child attachment induced by synchronization could be one of the possible explanations for such mood enhancement. Our hypothesis is also based on the finding that parent–child home-based music activities affect infants’ prosocial skills (Williams et al., 2015).

Regarding cognitive ability and brain development, several studies have reported that an earlier onset of musical training (i.e., ≤age 7 years) yielded greater cognitive and motor abilities (Watanabe et al., 2007; Bailey and Penhune, 2012). A review of the influence of musical training on child development concluded that musical training within a sensitive period affects children’s brain structure and cognitive abilities (Penhune, 2011). For example, the percentage of correct answers on an absolute-pitch test increased with age but plateaued at 7-years-old (Trainor, 2005; Miyazaki and Ogawa, 2006). Later impacts of music training during childhood on cognitive abilities have also been reported. Adult musicians who began musical training earlier in life could perform a timed motor-sequence task better than those who began later in life, even though the total length of musical training was similar in both groups (Watanabe et al., 2007). Brain research also suggests that the anterior half of the corpus callosum of music performers who began musical training before 7-years-old is larger than that of individuals who began training after 7-years-old (Schlaug et al., 1995). A more recent study also suggested that musical training changes children’s brains (Habibi et al., 2018).

Nonetheless, despite the findings, it remains unclear whether the age of onset of group music lessons for very young children affects their later sociability. There is little research on the associations between later sociability and regular long-term group music lessons that commenced from a very early age. Furthermore, although laboratory experiments have examined the effects of relatively short periods of musical training on sociability, the effect of regular group music lessons in actual music schools has rarely been explored.

Thus, we focused on the following research question: what is the association between onset age of group music lessons and later levels of sociability in children aged younger than 7 years? Based on the following three factors, we hypothesized that children with a very early onset of musical training (since 1-year-old) will exhibit higher levels of sociability than will children with a later onset of musical training (since ages 2, 4, and 6 years).

First, prior to the study, we conducted a preliminary survey of the association between age of onset and extracurricular musical training or activity in non-music majors (see Appendix). Participants’ responses implied that musical experience from a very early age positively influenced their social skills during adulthood. This result encouraged us to test our hypothesis. Second, prior studies suggested that musical training (e.g., Ilari, 2016) and group music activities (e.g., Kirschner and Tomasello, 2010) influence sociability in children. Therefore, group music lesson with parents should induce multi-directional interactions such as teacher–child, parent–child, and peer–peer, which may enhance prosociality (e.g., Keller et al., 2014) and attachment that is associated with children’s social-cognitive skills (Edwards and Abad, 2016). Third, considering findings that a critical age period strongly positively influences both musical and non-musical abilities (e.g., Miyazaki and Ogawa, 2006), the effects of onset of music activity and levels of sociability are probable.

Examining children with a maximum lesson period of 5 years may provide an additional aspect of the effects of group music lessons, since previous findings highlighted the impact of relatively short-term music activities during early childhood or the impact of musical education experience/non-experience. Owing to such long lesson periods, it was difficult to establish an experimental setting in which a random assignment design could be applied (Hille and Schupp, 2015). Thereby, we investigated participants who already took lessons. Furthermore, we investigated reasons for taking music lessons, parents’ levels of sociability, as well as children’s and parents’ personality traits. This was based on Sloboda and Howe’s (1991) finding that parents generally decide whether young children (in this case, toddlers) take music lessons, and that children’s personality may be influenced by parents’ personality traits due to a link between genetic factors and personality traits (Bouchard and Loehlin, 2001).

## Materials and Methods

### Design

A cross-sectional study was conducted to examine levels of sociability in groups of children aged 4–5 and 6–7 years with formal musical training through regular attendance at group music lessons in music schools. Ratings on psychometric instruments were dependent variables, while the categories of age of onset of children’s musical lessons were independent variables.

### Participants

We examined the levels of sociability of the children in the same age groups whose onset of the lessons varied. The same age groups of children included the first grade of elementary school (aged 6–7 years) and the equivalent to mid-year of kindergarteners (aged 4–5 years). Hereinafter, they are called elementary schoolers and preschoolers, respectively. Each age group involved children who started the lessons at different ages. Their group codes are in Table 1.

**Table 1:** Number of participants in each group.

Age of onset	*n*	Mean duration of lesson (months)	Codes of groups
**Elementary-schoolers (*M*_age_ = 82.7 months)**			
From age 1 year	31	65.4	Very-early starters
From age 2 years	39	53.4	Early starters
From age 4 years	40	29.9	Mid starters
From age 6 years	35	4.9	Late starters
**Preschoolers (*M*_age_ = 58.5 months)**			
From age 1 year	41	44.2	Very-early starters
From age 2 years	40	30.1	Early starters
From age 4 years	45	3.6	Mid starters


The ages that children began group lessons were either 1, 2, 4, or 6 years. Hereinafter, they are called as very-early starters, early starters, mid starters, and late starters, respectively. Parents (*N* = 276) responded to the questionnaire. We analyzed 271 total responses (260 mothers, 9 fathers, 1 grandmother, and 1 grandfather), which consisted of 145 elementary schoolers and 126 of preschoolers (86 boys, 184 girls, and 1 unknown; see Table 1).

Children engaged in group music lessons at the Yamaha Music School in the capital area (Tokyo and Kanagawa prefecture). Tokyo and Yokohama are the most populous cities in Japan, and they are adjacent to prefectures in the capital area. To avoid negative effects of this survey on the music school operation, personal data such as parents’ income and academic background were not collected; however, we predicted that familial socioeconomic and regional backgrounds would be similar to a certain degree: parents’ level of educational consciousness was high (as described later), parents selected lessons for which a fee was charged, and they lived in neighboring areas.

### Group Lessons

We conducted the survey at a private musical school—the Yamaha Music School. Contents of the group lessons were as follows.

#### Yamaha Music School

The Yamaha Music School is the largest music school that was developed by a private company in Japan, comprising approximately 378,000 students in Japan and 199,000 students internationally in 2017. Children from infants to high-school students are the main targets. The main courses are modest-sized group lessons as described below. The philosophy of the school was as follows: “To develop the musical sense that all people possess, and to develop their skills to create music themselves, perform, and enjoy music, in order to spread the joy of music” (Yamaha Music Foundation, 2016).

#### Contents of Group Lessons

A teacher, children, and parents participated in the lessons. Both children and parents, except for elementary schoolers (described below), attended a lesson together, which was conducted by a teacher. The age that children began lessons differed among groups in the present study: from around 1-year-old, 2-years-old, 4-years-old, or 6-years-old (first grade). The number of participants in each group is listed in Table 1. The duration of the lessons was 40 min per session. The shortest duration of lesson experience approximated 3 months because we conducted the survey from August to January and all lessons began in May (in the same year of the survey).

Contents of the lessons for each age group were as follows. All were group lessons, in which teachers often used a piano, electronic organ (Electone), or percussion. For children aged 1–3 years, child–parent pairs participated in the group lessons in an open space. The times of the lessons per month for each age were as follows: two times per month for 1-year-old, two or three times per month for 2-years-old, and three times per month for 3-years-old. The maximum capacity of the lesson was eight parent–child pairs. In this period, parents watched, helped, and facilitated their children more than they did at a later age. The content of the lessons was as follows: singing, appreciating music, and playing an instrument (such as percussion, a compact glockenspiel, and so on). Children aged 4 years also engaged in musical expression via physical activity.

For children aged 4 and 6 years, parent–child pairs participated in group lessons 40 times per year (i.e., 3–4 times per month), for 60 min per session. The maximum capacity of lessons was ten parent–child pairs (usually 4–6 parent–child pairs). Each pair sat with access to an electronic organ (Electone). The content of the lessons was as follows: comprehensive musical study using repertoire pieces, integrated study using musical pieces in the repertoire (listen, sing, play, and read), solfeggio, playing easy accompaniment music, playing in an ensemble, and so on. The main instrument used in the lessons was an electronic organ (Electone) or piano.

For children aged 6 and 7 years, only children (i.e., without parents) participated in group lessons three times per month, for 60 min per session. The maximum capacity of each lesson was ten children (4–6 children usually participated in each lesson). The content of the lessons was as follows: musical study of harmony, ensemble performance, singing, reading musical notation, musical grammar, and playing solo repertoire pieces using an electronic organ (Electone) or piano. The main instrument used in the lessons was an electronic organ (Electone) or piano.

### Psychometric Instruments

To examine children’s sociability, we used six existing scales rated by parents or adults close to the child. The present study reported both significant (*p* < 0.05) and marginally significant (*p* < 0.1) results.

#### Empathy

To assess empathy, we used the 23-item scale developed by Dadds et al. (2008) that measures children’s cognitive, affective, and total empathy. Responses are measured with a 9-point scale from -4 (*strongly disagree*) to 4 (*strongly agree*). Since empathy is related to prosocial behavior (Eisenberg and Miller, 1987), these items were selected. We translated the English questionnaires into Japanese verbatim. To check the translated version of the scale, Cronbach’s α coefficient was calculated. Cronbach’s α coefficient were as follows: 0.61 (cognitive empathy), 0.70 (affective empathy), and 0.78 (total empathy).

#### Personality

To assess personality traits, we used the Big Five Scales for Children developed by Deal et al. (2007), which incorporates five domains (neuroticism, extraversion, openness, agreeableness, and conscientiousness) and 15 subscales (achievement orientation, activity level, antagonism, compliance, consideration, distractibility, fearfulness/insecurity, intelligence, negative affect, openness to experience, organization, positive emotions, shyness, sociability, and strong-willed). Since extraversion in the five-factor theory of personality involves social skills (McCrae and Costa, 1999), personality was investigated.

The scale consisted of 50 items on a 7-point scale from 1 (*much less than the average child or not at all*) to 7 (*much more than the average child*). Each subscale consisted of 3 or 4 items. We translated the English questionnaires into Japanese. To check the translated version of the scale, we calculated Cronbach’s α coefficients. Coefficients for each of the factors were as follows: 0.57 (achievement orientation), 0.90 (activity level), 0.64 (antagonism), 0.45 (compliance), 0.77 (considerate), 0.58 (distractible), 0.55 (fearful/insecure), 0.78 (intelligent), 0.83 (negative affect), 0.71 (openness to experience), 0.68 (organized), 0.67 (positive emotions), 0.80 (shy), 0.83 (sociable), and 0.76 (strong-willed). Cronbach’s αs by subcategories were 0.68 (neuroticism), 0.74 (extraversion), 0.67 (openness), 0.75 (agreeableness), and 0.69 (conscientiousness). Several subscales showed low coefficients; however, their values on the original version were adequate (0.67–0.87). We employed these values since a small number of items could lead to Cronbach’s α being underestimated.

#### Social Skills

To assess social skills, we used the Social Skills of Preschoolers test developed by Takahashi et al. (2008), which incorporates a three-factor structure: cooperation (e.g., supporting others), self-control (e.g., coping with conflicts), and assertion (e.g., expressing emotions). The scale consisted of 30 items measured with a 3-point scale (from *not at all* to *always*). This scale was developed referring to existing scales (e.g., Frankenburg et al., 1992). Cronbach’s α of the Takahashi and colleagues’ study ranged from 0.89 to 0.98. Parents provided ratings for this scale, although it was originally intended to be rated by nursery teachers.

#### Social Adaptability

To measure social adaptability, we used the Asahide Social Adaptive Skills Test (Asahide-gakuen-kyoiku-kenkyu-sho, 2012). This scale was developed referring to previous studies (e.g., Sparrow et al., 1984). We employed subsets of the scale, considering our aim and the desire to avoid an excessively long survey. Sixty-nine items were employed. Parents rated them on a 2- or 3-point scale: 0 (*can not do or never do*), 1 (*can do in some cases or can do conditionally*), and 2 (*can do or could do*). Option “1” could be chosen in 8 of the 69 items. The domains were as follows: listening to others’ talk, asking questions, telling someone one’s experience, expressing denial and demands, interest and empathy for other people, conversation and communication, getting along with friends, cooperative relationships, following the rules for group play, appropriate manners, consideration for others, and control of emotions and behavior.

#### Other Measures

To assess the personality traits and social skills of parents, we used the Japanese version of the Ten Item Personality Inventory (Oshio et al., 2012) and the scale for social skills developed by Kikuchi (1988). The Ten Item Personality Inventory consisted of 10 items measured with a 7-point scale from 1 (*not at all*) to 7 (*strongly agree*). The original scale was developed by Gosling et al. (2003). Each subscale consisted of 2 items. The scale for social skills consisted of 18 items measured with a 5-point scale from 1 (*never*) to 5 (*always*).

Participants also reported basic personal attributes (e.g., age). We also asked parents why they enrolled their child in music lessons. Reasons were divided into four categories: (1) Child was interested in music or instruments, or often sang (“child’s interest in music”); (2) Child wanted to participate in lessons (“child’s desire to participate”); (3) Parents thought that lessons would be beneficial for their child’s education or development (“for child’s education or development”); and (4) Other people recommended that the parent should enroll their child in lessons (“influence of others”). Open-ended responses and multiple answers were allowed.

### Procedure

The survey was conducted by the authors at the Yamaha Music Foundation. The questionnaires were distributed to parents who took music lessons and retrieved later. A 1000-yen voucher (approximately 8–10 US dollars or 7–9 euros) was given to each participant. Informed consent was given along with the questionnaires. Questionnaires were anonymous and personal information was not collected. Participation was not mandatory. If participants did not wish to provide consent, they submitted a blank questionnaire without answering. This study was approved by an ethics committee at Soai University, Japan.

## Results

### Empathy

**FIGURE 1:**
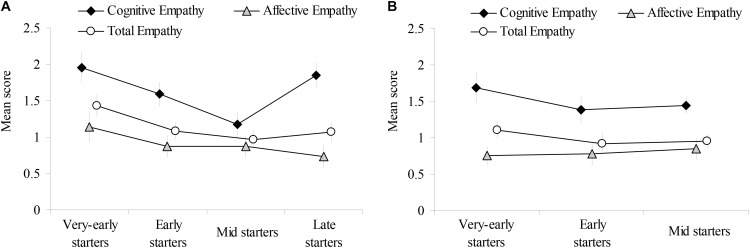
Mean scores of children’s empathy. Error bars represent standard errors. **(A)** Results of the elementary schoolers; **(B)** Results of preschoolers.

Figure 1 shows the average empathy ratings of each group. To compare ratings among groups, an analysis of variance (ANOVA) was conducted (Table 2; codes of groups were shown in Table 1). For elementary schoolers, the cognitive empathy scores of very-early starters and late starters were significantly higher than that of mid starters. In addition, the total empathy score of very-early starters was marginally higher than that of mid starters. For preschoolers, empathy scores did not differ among groups (*ps* > 0.1), although the cognitive empathy score and total empathy of very-early starters were high. In sum, the first-grade children, who had taken lessons since they were 1-year-old, showed high cognitive and total empathy. Children who had only just started lessons in the first grade also showed high cognitive empathy.

**Table 2:** Results of the ANOVA for cognitive empathy, affective empathy, and total empathy and *post hoc* tests.

	Elementary schoolers			
	
	*F*	*p*	η^2^	*Post hoc* test (Tukey)
CE	3.794	0.012	0.075	Very-early starters > mid starters (*p* = 0.015) Late starters > mid starters (*p* = 0.040)
AE	0.924	0.431	0.019	
TE	2.258	0.084	0.046	Very-early starters > mid starters (*p* = 0.065)


*All *df*s for the elementary schoolers were [3, 140]. CE, cognitive empathy; AE, affective empathy; TE, total empathy; ANOVA, analysis of variance.*

### Personality

To examine personality traits of each group, an ANOVA was conducted (Table 3). Regarding Big Five personality traits, the extraversion and openness of late starters of elementary schoolers were marginally significantly higher than those of early starters. Openness of very-early starters was marginally significantly higher than that of early starters. No significant or marginally significant differences existed among groups of preschoolers (*ps* > 0.1).

**Table 3:** Results of ANOVA and *post hoc* tests for personality traits.

	Elementary schoolers				Preschoolers			
		
	*F*	*p*	η^2^	*Post hoc* test (Tukey)	*F*	*p*	η^2^	*Post hoc* test (Tukey)
**Domain**								
Extraversion	2.620	0.053	0.053	Late starters > early starters (*p* = 0.056)				
Openness to experience	3.325	0.022	0.066	Very-early starters > early starters (*p* = 0.067) Late starters > early starters (*p* = 0.055)				
Agreeableness	2.145	0.097	0.044					
**Subscale**								
Achievement orientation	3.084	0.029	0.062	Late starters > early starters (*p* = 0.081)				
Activity level	4.481	0.005	0.087	Very-early starters > early starters (*p* = 0.015) Mid starters > early starters (*p* = 0.074) Late starters > early starters (*p* = 0.010)				
Compliance	2.330	0.077	0.047	Late starters > mid starters (*p* = 0.086)	2.454	0.090	0.039	Very-early starters > early starters (*p* = 0.083)
Consideration	3.376	0.02	0.067	Late starters > early starters (*p* = 0.044) Late starters > mid starters (*p* = 0.023)				
Fearfulness/insecurity					2.565	0.081	0.040	Mid starters > very-early starters (*p* = 0.065)
Intelligence	2.357	0.074	0.048	Very-early starters > early starters (*p* = 0.073)				
Negative affect	2.332	0.077	0.047	Very-early starters > early starters (*p* = 0.050)				
Positive emotions	2.694	0.048	0.054	Late starters > early starters (*p* = 0.064)				


*Only significant and marginally significant results are described. All *df*s for the first grade were [3, 141], and all *df*s for the middle grade of kindergarten were [2, 122]. ANOVA, analysis of variance.*

Regarding subscales, for the elementary schoolers, activity level of very-early starters was significantly higher than those of early starters. Intelligence and negative affect of very-early starters were marginally higher than those of early starters. Activity level and consideration of late starters was significantly higher than those of early starters. Achievement orientation and positive emotions of late starters were marginally higher than those of early starters. Furthermore, compliance and consideration of late starters were higher than those of mid starters. For preschoolers, compliance of very-early starters was higher than that of early starters, while fearfulness/insecurity of very-early starters was lower than that of mid starters.

### Social Skills

For both elementary schoolers and preschoolers, no items provided statistical difference concerning the Social Skills of Preschoolers (*ps* > 0.1).

No items of the Asahide Social Adaptive Skills Test differed statistically among elementary schoolers (*ps* > 0.1). For preschoolers, the scores of “interest and empathy for other people” and “conversation/communication” of very-early starters were significantly higher than those of early starters, while the scores of “talking about experiences,” “conversation/communication” of very-early starters were significantly higher than those of mid starters (Table 4). The score of “manners” among very-early starters was marginally significantly higher than that of mid starters. In sum, preschoolers who had taken lessons from 1-year-old showed higher ratings of some adaptive social skills than did those in the other groups.

**Table 4:** Results of ANOVA and *post hoc* tests for social adaptability.

	Preschoolers			
	
	*F*	*p*	η^2^	*Post hoc* test (Tukey)
Telling someone one’s experience	2.978	0.055	0.046	Very-early starters > mid starters (*p* = 0.042)
Interest and empathy for other people	2.992	0.054	0.046	Very-early starters > early starters (*p* = 0.046)
Conversation and communication	4.281	0.016	0.065	Very-early starters > early starters (*p* = 0.037) Very-early starters > mid starters (*p* = 0.029)
Appropriate manners	2.53	0.084	0.040	Very-early starters > mid starters (*p* = 0.067)


*Only significant and marginally significant results are described. All *df*s for the middle grade of kindergarten were [2, 123]. ANOVA = analysis of variance.*

### Parents’ Personality and Social Skills

Regarding parents’ personality and social skills, the openness of the parents of elementary schoolers was significant [*F*(3,140) = 5.272, *p* = 0.002, η^2^= 0.101]. Multiple comparisons showed that the openness of the parents of late and mid starters was significantly or marginally significantly higher than that of the parents of very-early and early starters (late starters > very-early starters, *p* = 0.071; mid starters > early starters, *p* = 0.080; late starters > early starters, *p* = 0.001). The other items did not yield significant differences among groups.

### Reasons for Participation in Lessons

**FIGURE 2:**
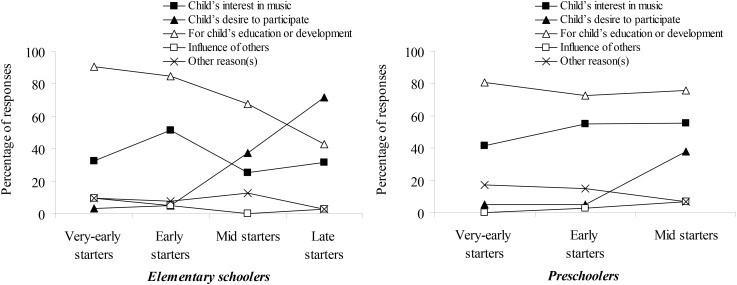
Reasons for participating in lessons in each group.

Figure 2 shows the percentage of each response for reasons for participating in lessons. For the elementary schoolers, the most frequent response, except for those by late starters, was the item, “parents think that music lessons could contribute to their child’s education or development.” In contrast, the older the onset age, the less frequent this response became in mid and late starters. Instead, the item, “child wanted to participate in music lesson” was selected more frequently by these groups.

Furthermore, for early starters, the frequency of the item, “child was interested in music or instrument, or frequently sang” was higher than it was in the other groups. In the preschoolers, there were tendencies toward selecting “for child’s education or development” for children who started lessons at age 1 or 2 years, and an increase in choices of “child’s wanting to participate” in children who started lessons in mid starters. The results suggested that while at an early age taking lessons depended on parents’ will and initiative, at a later age, taking lessons was more related to children’s desire.

## Discussion

We investigated how the age at which group music lessons began is associated with children’s levels of sociability by focusing on empathy, personality, and social skills in children aged 6–7 and 4–5 years. We hypothesized that a very early onset of musical training would be associated with later sociability. The results of our cross-sectional survey suggested that (1) the empathy of very-early starters of the elementary schoolers was higher than that of mid starters; (2) the scores of communication-related items of very-early starters of preschoolers were higher than those of other age groups; and (3) the empathy scores of late starters of elementary schoolers were high, as were their extraversion scores. Consequently, these results supported our hypothesis that children who experience a very early onset of musical training (from 1-year-old) will exhibit higher levels of sociability than will those of later onsets; however, of note, the late starters among the elementary schoolers also showed high levels of sociability.

First, the sociability-related scores of children who started group music lessons very early were superior to those who began lessons later. This tendency was especially significant in elementary schoolers. The empathy of the very-early starters of elementary schoolers was higher than that of children whose onset was later than 1-year-old. Since empathy has been reported to be related to social skills (Eisenberg and Miller, 1987), starting group music lessons at a very-early age is likely associated with later sociability. In addition, the very-early starters of preschoolers yielded significantly higher scores for communication-related items of social adaptation (telling someone one’s experience, interest and empathy for other people, and conversation and communication) than did early or mid-starters of the same age.

Our results suggest a novel perspective: the impact of regular group music lessons from a very early age on later levels of sociability. Prior findings had only focused on the effects of music activities during early childhood over a relatively short period or the effect of musical education experience/non-experience. Simultaneously, the results support previous studies showing that music activities enhance children’s sociability (Hallam, 2010; Ilari, 2016), even infants (Gerry et al., 2012; Cirelli et al., 2014; Williams et al., 2015). Similarly, the results are consistent with findings that musical activities facilitate children’s sympathy and prosocial behavior (Schellenberg et al., 2015), specific prosocial behaviors (Kirschner and Tomasello, 2010), empathy (Rabinowitch et al., 2012), and social skills (Hallam and Prince, 2000).

Why did an earlier onset of the group music lessons promote increased sociability? Multifaceted joint musical activities with other group members—peers, parents, and a teacher—may be one possible explanation. These interactions were expected to lead to high sociability among children, since synchronization facilitates infants’ prosocial behavior (Carpenter et al., 2013; Cirelli et al., 2014), empathy (Behrends et al., 2012), cooperative skills (Valdesolo et al., 2010), and compassion and altruistic behavior (Valdesolo and DeSteno, 2011). In addition, given that joint musical performance requires social skills in performers (Keller, 2014; Kawase, 2015; Volpe et al., 2016), interactions with members in the lessons could foster children’s sociability.

Parent–child musical interactions is another likely explanation for the enhanced sociability. In the lessons, simultaneous parent–child musical activities, in which accompanying parents supported their children during singing to music, playing instruments, and dancing to music, likely generated attachment. As attachment produced by parent–child synchronization increases (Isabella and Belsky, 1991; Creighton, 2011; Edwards, 2011), children’s social-cognitive skills are in turn enhanced (reviewed in Thompson, 2008). Since younger children require more support from their parents, attachment might increase in the younger classes as compared to the older classes. Of course, children who did not take lessons at 1-year-old can still engage in activities that will induce attachment; however, joint musical activity that requires frequent synchronization or coordination with members might be a stronger means to enhance parent–child attachment. This is supported by the finding that parent–child coordination during musical activity was shown to be beneficial for good relationships, regardless of controlling for other types of parent–child activities (Wallace and Harwood, 2018).

Another possible explanation for the effects of earlier onset of music activity is brain plasticity—children are within a sensitive period, as reflected in areas such as auditory responses and absolute pitch (Trainor, 2005). The present results for children’s empathy showed that the earlier the onset of the lessons, the higher children’s empathy became. This tendency is similar to that of absolute pitch acquisition (Miyazaki and Ogawa, 2006). Given that social skills involve cognitive abilities (Riggio, 1986), it can be inferred that cognitive development is somewhat associated with sociability. This result is also consistent with the findings that the sensitive period in musical training affects children’s brain structure and cognitive ability (Penhune, 2011), and that earlier music training yielded better scores on timed motor-sequence tasks in adult musicians with similar overall musical training (Watanabe et al., 2007; Bailey and Penhune, 2012).

The impact of music lessons from a very young age is also supported by the U-shaped relationship between the onset of musical experience and social skills in non-music majors in our preliminary survey (see Appendix); however, the coefficients of determination were low. We found that social skills were associated with the age at which musical experience began, rather than total amount of musical experience. Further, when participants began music lessons before 7-years-old, earlier onset was associated with improved social skills.

Second, contrary to our hypothesis, we found an unexpected result in elementary schoolers who had just commenced the group music lessons. The level of empathy of late starters was high. In addition, their extraversion was marginally higher than was that of early starters. In addition, their levels of positive emotion and consideration (within the subscale of agreeableness) were high. This result appears to contradict the result that starting group music lessons from very early childhood was associated with high levels of sociability.

One possible explanation for this contradictory finding is that children’s aptitude for group music lessons in which they attend a course with other children requires social interactions and social skills (Keller, 2014; Kawase, 2015; Volpe et al., 2016). As Figure 2 indicates, as children age, their desire to participate increases. In addition, from the first grade of elementary school (i.e., aged 7 years), students at the Yamaha Music School can select between one-on-one or group lessons; the children in the present study eventually took up group music lessons despite the availability of one-to-one lessons. Since elementary schoolers could understand the content of group lessons, children who engage in group music lessons may be inclined to prefer group activity to solo activity, or not to hesitate to participate in group activities. Therefore, children who voluntarily participated in lessons at a later age might already be sociable. This is supported by findings that music majors who like or are skilled at ensemble performance show greater social skills (Kawase, 2015), agreeableness, and extraversion (Kawase, 2016). Furthermore, this result is in accordance with our preliminary survey showing that after age 7 years, later onset of musical activity led to greater social skills. As the triggers of music-lesson participation in the first grade of elementary school show, along with children’s growth, voluntary motivation may be a strong reason for participating in musical activities, such as club activities at school. More strict studies would be useful to investigate relationships between such motivation of the musical activity and sociability in childhood.

Finally, despite the role of genetic factors in personality traits (Bouchard and Loehlin, 2001), the effects of parents’ social skills and personality traits did not differ among groups, except for openness. Therefore, parents whose sociability was higher did not necessarily affect whether they enrolled their children in music lessons at a younger age.

## Conclusion

Our results imply that participation in regular group music lessons could be a reasonable means for promoting children’s levels of sociability, particularly for very young children whose social behaviors are malleable (Brownell, 2013). Having lessons 2–4 times per month is probably not too much music activity for children and their parents; thus, such effects of sociability also bear out very young children’s parents’ expectations for their sociability in group music activity (Pitt and Hargreaves, 2017).

In future studies, it will be necessary to investigate whether the present study outcomes are applicable to children older than 7 years. We focused younger children, in whom the effects of music lessons are inclined to appear because of drastic brain plasticity (Schlaug et al., 1995; Penhune, 2011). Furthermore, more strict socio-economic factors such as diverse local areas or incomes should be examined; although, we collected data from similar areas to avoid the influence of such factors. In addition, it will be useful to examine what factor—onset age or lesson duration—affects sociability; therefore, future studies should also examine children who commenced group music lessons at an early age but quit halfway through. From a wider perspective, it would also be useful to explore whether the relationships we found between the age of onset of music lessons and later sociability are applicable to diverse types of musical lessons for preschoolers; for example, music classes for infants based on Suzuki methods that were reported to influence sociability (Gerry et al., 2012). In addition, comparison between one-to-one and group lessons can reveal whether the numbers of participants in the lesson is associated with levels of sociability. Finally, further studies should confirm children’s levels of sociability through their behaviors, as sociability was only evaluated using parent rating scales in the present study.

## Author Contributions

SK designed the study, analyzed and interpreted the data, and drafted the manuscript. JO also planned the study and conducted the survey. SO and TH collected data of the preliminary survey.

## Conflict of Interest Statement

SK and JO were employed by the Yamaha Music Foundation. SO was employed by the Yamaha Corporation. The remaining author declares that the research was conducted in the absence of any commercial or financial relationships that could be construed as a potential conflict of interest.

## Figures and Tables

**FIGURE A1:**
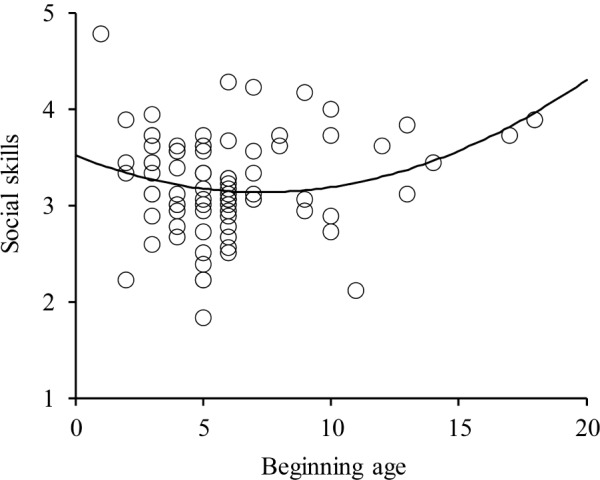
Relationship between beginning age of musical experience and social skills. Curve shows the results of a quadratic regression analysis.

## Appendix: Supplemental Survey

To examine the relationship between extracurricular musical experience (e.g., club activity in school or piano lessons) and levels of sociability, we conducted a preliminary survey of the sociability of adults and the onset age of their musical experience and their overall musical experience. Participants were non-music majors, because music majors generally continue to take lessons into adulthood—which means that their total duration of lessons is greater and their onset age of music lessons is typically earlier than that of other individuals.

### Participants

Eighty-eight non-music majors who had musical experience responded to the questionnaire (*M_age_* = 19.9 years, *SD* = 1.8 years). The average age that musical activity began was 6.2 years (*SD* = 3.4 years), and the average duration from music lessons was 8.1 years (*SD* = 4.4 years). The most common instrument played was the piano (*n* = 79).

### Procedure and Data Analysis

College students participated in the survey, which was conducted at Osaka University, Osaka Seikei University, Osaka University of Human Science, Kobe Yamate College, and Kobe Shinwa Women’s University by the experimenter and lecturers. Participants could take as much time as necessary to complete the survey (the average time was 20–30 min). The survey included other scales intended for another study. Informed consent was obtained via the following statement that was a part of the questionnaire. Questionnaires were anonymous and personal information was not collected. Participation was not mandatory. Unanswered questionnaires or questionnaires not returned was deemed as not consenting to participate.

### Psychometric Instruments

#### Social Skills

To examine holistic social skills, we used Kikuchi’s Social Skills scale (Kikuchi, 1988). It consists of items such as “Do your conversations flow while talking with other people?” This 18-item scale is measured using a 5-point rating scale, which was generated based on Goldstein et al. (1980) work with adolescents. The validity of this scale has been supported by many studies in Japan (as reviewed in Kikuchi, 2004).

#### Empathy

To gauge empathy, we used Sakurai’s Japanese multidimensional scale of empathy (Sakurai, 1988), which is based on Davis’s (1983) scale, and whose factors are the same as Davis’; i.e., perspective taking, fantasy, empathic concern, and personal distress. It consisted of 28 items measured with a 4-point scale.

#### Personality Traits

To assess personality traits, we used the short form of the Japanese Big Five Scale, which consists of 29 items measured with a 7-point

scale (Namikawa et al., 2012). We used the brief version to reduce the burden on participants. In accordance with item response theory, this scale was created by abbreviating Wada’s (1996) scale, whose 60 items were based on Gough and Heilbrun’s (1983) work.

### Results

The results of linear and quadratic regression analyses showed that non-standardized *B*-coefficients were significant for the onset of musical experience as a predictor of social skills, and duration of musical experience as a predictor of perspective taking (Table A1). Both social skills and perspective taking were significant only in a quadratic regression. Age of onset predicted a greater amount of variation in social skills than did duration of perspective taking, although the coefficient of the former was not as large. Accordingly, before the age of 7 years, social skills tended to improve with an earlier onset of musical experience; however, after the age of 7 years, social skills tended to be higher with a later onset of musical experience (Figure A1).

**Table A1:** Results of regression analysis of non-music majors.

Musical experience	Scale	*B*^2^ (×10^-3^)	*B* (×10^-2^)	*Const*.	Adjusted *R*^2^	Bottom of the curve
Beginning age	Social skills	7.21^∗^	-10.48^†^	3.52^∗∗^	0.062	7.27
Duration	Perspective taking	5.73^∗^	-9.56^†^	2.98^∗∗^	-0.012	8.35

*^†^*p* < 0.1, ^∗^*p* < 0.05, ^∗∗^*p* < 0.01.*
